# Tubulin Cytoskeleton Organization in Cells of Determinate Nodules

**DOI:** 10.3389/fpls.2022.823183

**Published:** 2022-04-26

**Authors:** Anna B. Kitaeva, Artemii P. Gorshkov, Pyotr G. Kusakin, Alexandra R. Sadovskaya, Anna V. Tsyganova, Viktor E. Tsyganov

**Affiliations:** ^1^Laboratory of Molecular and Cellular Biology, All-Russia Research Institute for Agricultural Microbiology, Saint Petersburg, Russia; ^2^Faculty of Biology, Saint Petersburg State University, Saint Petersburg, Russia; ^3^Saint Petersburg Scientific Center RAS, Saint Petersburg, Russia

**Keywords:** legume–rhizobial symbiosis, microtubules, symbiosome, bacteroid, determinate nodules, *Glycine* spp., *Phaseolus vulgaris*, *Lotus japonicus*

## Abstract

Plant cell differentiation is based on rearrangements of the tubulin cytoskeleton; this is also true for symbiotic nodules. Nevertheless, although for indeterminate nodules (with a long-lasting meristem) the organization of microtubules during nodule development has been studied for various species, for determinate ones (with limited meristem activity) such studies are rare. Here, we investigated bacteroid morphology and dynamics of the tubulin cytoskeleton in determinate nodules of four legume species: *Glycine max*, *Glycine soja*, *Phaseolus vulgaris*, and *Lotus japonicus*. The most pronounced differentiation of bacteroids was observed in *G. soja* nodules. In meristematic cells in incipient nodules of all analyzed species, the organization of both cortical and endoplasmic microtubules was similar to that described for meristematic cells of indeterminate nodules. In young infected cells in developing nodules of all four species, cortical microtubules formed irregular patterns (microtubules were criss-crossed) and endoplasmic ones were associated with infection threads and infection droplets. Surprisingly, in uninfected cells the patterns of cortical microtubules differed in nodules of *G. max* and *G. soja* on the one hand, and *P. vulgaris* and *L. japonicus* on the other. The first two species exhibited irregular patterns, while the remaining two exhibited regular ones (microtubules were oriented transversely to the longitudinal axis of cell) that are typical for uninfected cells of indeterminate nodules. In contrast to indeterminate nodules, in mature determinate nodules of all four studied species, cortical microtubules formed a regular pattern in infected cells. Thus, our analysis revealed common patterns of tubulin cytoskeleton in the determinate nodules of four legume species, and species-specific differences were associated with the organization of cortical microtubules in uninfected cells. When compared with indeterminate nodules, the most pronounced differences were associated with the organization of cortical microtubules in nitrogen-fixing infected cells. The revealed differences indicated a possible transition during evolution of infected cells from anisotropic growth in determinate nodules to isodiametric growth in indeterminate nodules. It can be assumed that this transition provided an evolutionary advantage to those legume species with indeterminate nodules, enabling them to host symbiosomes in their infected cells more efficiently.

## Introduction

Legumes have a symbiotic relationship with rhizobia through the formation of nitrogen-fixing nodules. Nodule formation involves different molecular-genetic and cellular mechanisms, one of which is cytoskeleton reorganization. In plants, the tubulin cytoskeleton is involved in various processes of cell development and function (Kost et al., 1999). Endoplasmic microtubules are involved in cell division (Li et al., 2015), organelle movement, and intracellular transport (Peña and Heinlein, 2013). At the same time, cortical microtubules (underlying the plasma membrane) are involved in cell wall formation and determine the shape of the cell (Wasteneys, 2004; Paradez et al., 2006; Bashline et al., 2014).

It has been clearly demonstrated that active cytoskeleton rearrangements are required at different stages of nodule development (Timmers, 2008; Genre and Timmers, 2019; Tsyganov et al., 2019). Symbiotic nodules can be subdivided into two main types: indeterminate and determinate (Hirsch, 1992). The nodules of the first type are characterized by a prolonged functioning of the meristem, while in nodules of the second type, the meristem activity is transient. Differences in the activity of meristems lead to differences in the structure of nodules. Histological zonation is characteristic of indeterminate nodules, while it is absent in determinate ones (Guinel, 2009). As a result, indeterminate nodules are characterized by an elongated shape, while determinate nodules are spherical.

The mechanisms of organogenesis of determinate and indeterminate nodules also differ. Thus, in indeterminate nodules, the infection thread reaches the cells of the nodule primordium, which is formed as a result of the induction of cell division in the pericycle and inner cortex (Timmers et al., 1999). In determinate nodules, the infection thread reaches the outer (in *Glycine max* nodules) or middle (in *Lotus japonicus* nodules) cortex, which is located in the vicinity of the infected root hairs (van Spronsen et al., 2001). Involvement of the tubulin cytoskeleton during the early stages of infection thread growth has been demonstrated for nodules of both types (Timmers et al., 1999, 2007; Sieberer et al., 2005; Vassileva et al., 2005; Perrine-Walker et al., 2014). For *Medicago sativa* and *M. truncatula* nodules, reorientation of microtubules in cells of the inner cortex during nodule primordium formation has been described (Timmers et al., 1999). In mature indeterminate nodules, the important role of tubulin (Kitaeva et al., 2016) and actin cytoskeletons (Zhang et al., 2019) in infection thread and infection droplet development has been clearly demonstrated.

After being released from infection droplets into the cytoplasm of the plant cell, bacteria differentiate into bacteroids, while they are separated from the cytoplasm by the symbiosome membrane, forming organelle-like symbiosomes (Coba de la Peña et al., 2018; Tsyganova et al., 2018). In many legume species indeterminate nodules are characterized by strong morphological differentiation of bacteroids, which may even be terminal (Mergaert, 2020). At the same time, differentiation of bacteroids in determinate nodules is less morphologically pronounced. For example, bacteroids of *L. japonicus* strongly resemble free-living bacteria (Szczyglowski et al., 1998). A striking feature of bacteroids in determinate nodules is their ability to divide, which leads to the formation of symbiosomes containing several bacteroids. It has been shown for *G. max* that the juvenile symbiosome contains one bacteroid, while the mature symbiosome contains 2–4 bacteroids (Fedorova et al., 1999). Symbiosomes containing several bacteroids are also described for *Phaseolus vulgaris* (Cermola et al., 2000), *L. japonicus* (Szczyglowski et al., 1998), and *G. soja* (Temprano-Vera et al., 2018).

In both types of nodules, the number of symbiosomes in infected cells is crucially increased. The hosting of symbiosomes in infected cells recruits both tubulin (Kitaeva et al., 2016, 2021; Tsyganova et al., 2021) and actin cytoskeletons (Gavrin et al., 2015; Zhang et al., 2019). The detailed analysis of tubulin organization in the nodules of six different legume species forming indeterminate nodules revealed that endoplasmic microtubules are spread between symbiosomes and are organized in two main patterns: regular and irregular ones, corresponding to ordered or disordered distribution of symbiosomes (Kitaeva et al., 2016, 2021; Tsyganova et al., 2021). The regular pattern is common for infected cells in *M. truncatula* and *Galega orientalis* nodules, whereas the irregular one is a prerequisite for *Cicer arietinum* and *Pisum sativum* infected cells; finally, an intermediate pattern is characteristic of *Vicia sativa* and *Glycyrrhiza uralensis* infected cells. It has been suggested that the pattern of endoplasmic microtubules correlates with bacteroid size and shape (Kitaeva et al., 2021).

The accommodation of thousands of symbiosomes in the infected cell requires plant cell differentiation that is accompanied by a significant increase in cell volume in both determinate and indeterminate nodules (Tsyganova et al., 2018). It is known that the cortical tubulin cytoskeleton is involved in the determination of type of cell growth. Cortical microtubules that are oriented transverse to the cell growth axis determine anisotropic cell growth, whereas irregular orientation of cortical microtubules leads to isodiametric cell growth (Crowell et al., 2010; Hamada, 2014). In indeterminate nodules of six legume species the transverse orientation of cortical microtubules is a prerequisite for anisotropic growth of uninfected cells and colonized cells (with infection threads and droplets but without bacterial release). However, bacterial release leads to irregular orientation of cortical microtubules, which provides a possibility for isodiametric cell growth that allows a notable increase in cell size for the hosting of numerous symbiosomes (Kitaeva et al., 2016, 2021; Tsyganova et al., 2021).

It is necessary to note that involvement of the cytoskeleton, specifically microtubules, is poorly studied in determinate nodules; there is just one description of microtubular organization in *G. max* nodules (Whitehead et al., 1998). The aim of this study was to compare the organization of the tubulin cytoskeleton in four legume species that form determinate nodules, in order to compare the identified patterns with those in indeterminate nodules. Such an analysis should make it possible to reveal both general patterns and typical differences in the development of nodules of the two types.

## Materials and Methods

### Plant Material and Bacterial Strains

Seeds of *Glycine max* (L.) Merrill accession K-5892 Fiskeby V and *Glycine soja* Siebold & Zucc. accession K-11570 from the collection of the Federal Research Center N. I. Vavilov All-Russian Institute of Plant Genetic Resources (VIR) were kindly provided by Dr. Margarita Vishnyakova. Seeds of *Lotus japonicus* (Regel) K. Larsen accession B-129 “Gifu” (Jiang and Gresshoff, 1997) were kindly provided by Prof. Jens Stougaard, Aarhus University, Denmark. For *Phaseolus vulgaris* L. cv “Supernano” commercial seeds were used.

Seeds were sterilized in concentrated sulfuric acid for 5 min (*G. max*, *G. soja*, *P. vulgaris*) or 1 min (*L. japonicus*). After sterilization, the seeds were washed with sterile water 10 times and germinated at 28°C. Seeds of *G. soja* were scarified with a scalpel (Pueppke, 1983), prior to leaving for germination. Seeds of *L. japonicus* were germinated under full-light conditions. For inoculation, the following strains from the Russian Collection of Agricultural Microorganisms (All-Russia Research Institute for Agricultural Microbiology) were used: *Bradyrhizobium liaoningense* RCAM04656 (*G. max* and *G. soja*), *Rhizobium leguminosarum* bv. *phaseoli* RCAM2624 (*P. vulgaris*), and *Mesorhizobium loti* RCAM1804 (*L. japonicus*). Seedlings were inoculated with the corresponding rhizobia strain, using 1 ml of an aqueous suspension containing 10^7^–10^8^ cells per seed.

Plants were grown in sterile vermiculite wetted with nitrogen-free nutrient solution (Fåhraeus, 1957), in an MLR-352H growth chamber (Sanyo Electric Co., Ltd., Moriguchi, Japan) under controlled conditions: day/night, 16/8 h; temperature 21°C; humidity 75%; and illumination 280 μmol photons m^–2^ s^–1^). The nodules were harvested at days 10, 14, and 21 after inoculation.

### Microscopy

#### Electron Microscopy

The nodules for electron microscopy were harvested at day 21 after inoculation. The electron microscopy protocol was as previously described (Serova et al., 2018). Samples were embedded in Epon (Honeywell Fluka™, Fisher Scientific, Loughborough, United Kingdom). For transmission electron microscopy, ultrathin sections were cut on a Leica EM UC7 ultramicrotome (Leica Microsystems, Vienne, Austria). The nodule tissues were examined and photographed under a JEM-1400 transmission electron microscope (JEOL Corporation, Tokyo, Japan) at 80 kV.

For scanning electron microscopy, nodules were prepared as previously described (Tsyganova et al., 2021). The samples were observed in a Tescan MIRA3 LMU scanning electron microscope (Tescan, Brno, Czech Republic) at 9 kV.

#### Immunolocalization and Laser Scanning Confocal Microscopy

Visualization of microtubules was performed as previously described (Kitaeva et al., 2016). Some modifications that are necessary according to the specificity of every species (Kitaeva et al., 2018) were made. For each species, an optimally composed fixing solution was selected and used. Nodules of *G. max*, *G. soja*, and *P. vulgaris* were fixed in 1/5 microtubule stabilizing buffer (MTSB) (50 mM PIPES, 5 mM MgSO_4_⋅7H_2_O, 5 mM EGTA, pH 6.9) containing 3% formaldehyde, 0.25% glutaraldehyde, 0.3% Tween-20, 0.3% Triton X-100; *L. japonicus* nodules were fixed in 1/10 MTSB containing 3% formaldehyde, 0.25% glutaraldehyde, 0.3% Tween-20, 0.3% Triton X-100, 10% dimethyl sulfoxide. Nodule longitudinal sections were made using a microtome with a vibrating blade HM650V (Microm, Walldorf, Germany). Immunolocalization of the tubulin cytoskeleton, infection droplets, infection threads, and staining of nuclei and bacteria were performed as previously described (Kitaeva et al., 2021). Prior to staining with propidium iodide, sections of *G. soja* and *P. vulgaris* were incubated in RNAse A solution (Thermo Fisher Scientific, Waltham, MA, United States) at a dilution of 1:10 for 30 min at 28°C. Microtubule pattern analysis in nodule cells was performed using an LSM 780 laser scanning confocal microscope and ZEN 2012 software (Zeiss, Oberkochen, Germany). AlexaFluor 488 was excited at 488 nm, and fluorescence emitted between 499 to 543 nm was collected; Alexa Fluor 546 was excited at 561 nm, and emitted fluorescence between 568 and 572 nm was collected; propidium iodide was excited at 561, and emitted fluorescence between 606 and 677 nm was collected.

### Bacteroid Isolation

Bacteroids were isolated as previously described (Tsyganova et al., 2021). Briefly, 3-week-old nodules (five nodules for each species) were cut into pieces, digested by cellulase, and stained with propidium iodide. The length of 50 bacteroids of each species (165 bacteroids for *G. soja* due to the high variation in bacteroid length) was determined. Pairwise comparisons were conducted using Tukey’s range test.

### Free-Living Bacteria Visualization

Bacteria were visualized according to Tsyganova et al. (2021). Bacteria were heat-treated at 70°C and stained with propidium iodide. The length of 25 bacteria was determined. Pairwise comparisons were conducted using Tukey’s range test.

### Quantitative Analysis

For determining microtubule orientations in nodule cells of studied species a previously described method was used (Tsyganova et al., 2021). This involved converting confocal images to maximum intensity projections, their thresholding, and the use of MicroFilament Analyzer software (Jacques et al., 2013). Obtained frequencies of microtubule angles were classified relative to the longitudinal axis of the cell as axial (0–30, 150–180), oblique (30–60, 120–150), or transverse (60–120). Isolation and analysis of endoplasmic microtubules from z-stack confocal images were performed as described previously (Kitaeva et al., 2021; Tsyganova et al., 2021). Statistically significant differences in angle frequencies between the cell types were determined using Kruskal–Wallis test and Dunn’s *post hoc* test.

## Results

### Bacteroid and Symbiosome Morphology

**FIGURE 1:**
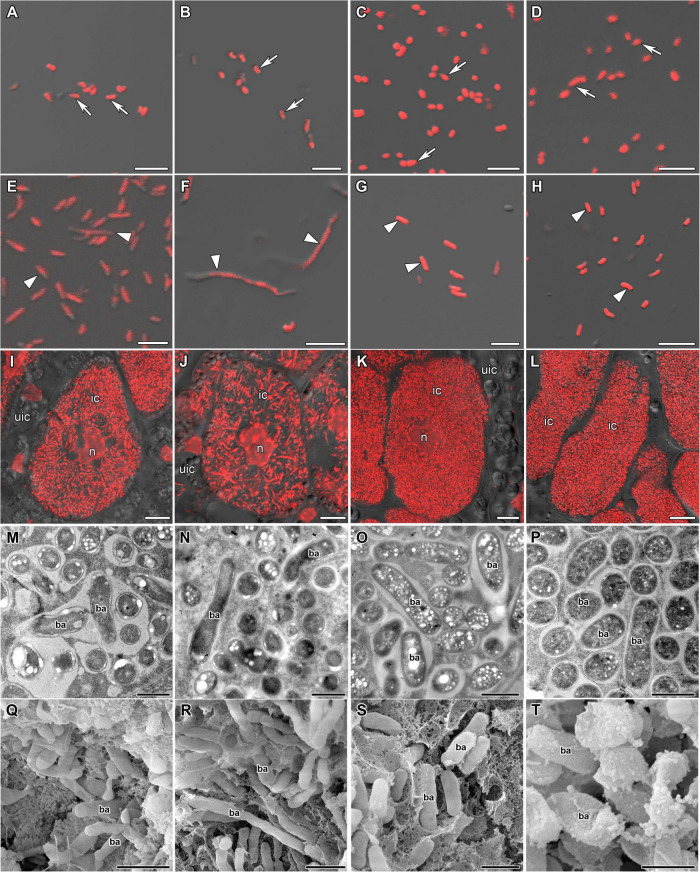
Morphology of bacteria, bacteroids, and infected cells in *G. max* **(A,E,I,M,Q)**, *G. soja* **(B,F,J,N,R)**, *P. vulgaris* **(C,G,K,O,S)**, and *L. japonicus* **(D,H,L,P,T)** nodules. ic, infected cell; uic, uninfected cell; n, nucleus; ba, bacteroid; arrows indicate bacteria, triangles indicate bacteroids. **(A–D)** Bacteria. **(E–H)** Bacteroids. **(I–L)** Symbiosome arrangement in infected cells of the nitrogen fixation zone. **(M–P)** Ultrastructure of an infected cell. **(Q–T)** Scanning electron microscopy of infected cells. **(A–L)** Merged images of differential interference contrast and red channel (DNA staining with propidium iodide (nuclei and bacteria)). Scale bar **(A–H)** = 5 μm, **(I–L)** = 10 μm, **(M–P)** = 1 μm, **(Q–T)** = 2 μm.

**FIGURE 2:**
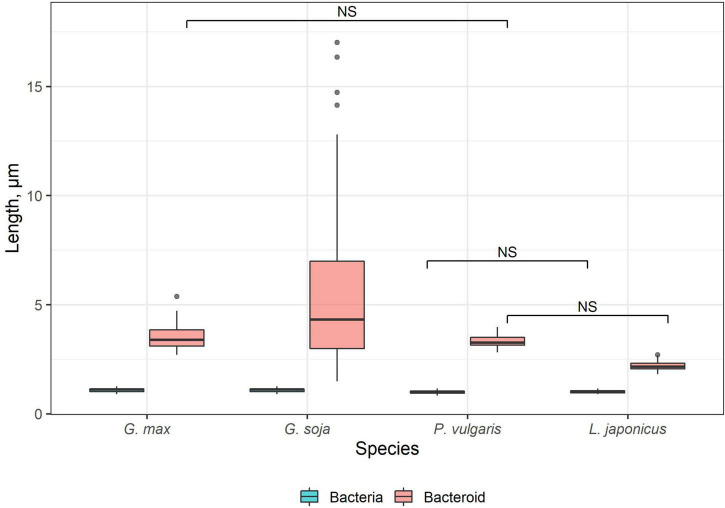
Length of free-living bacteria and bacteroids in nitrogen-fixing cells of *G. max*, *G. soja*, *P. vulgaris*, and *L. japonicus* nodules. Pairwise comparisons were conducted using Tukey’s range test. NS indicates not significant differences (*p* > 0.05); *n* = 50 (for *G. soja* n = 165 due to the high variation of bacteroid length).

Free-living bacteria of *B. liaoningense* RCAM04656, *R. leguminosarum* bv. *phaseoli* RCAM2624, and *M. loti* RCAM1804 were characterized by a similar shape (Figures 1A–D) and length of around 1 μm (Figure 2). At the same time, bacteroids in nodules of studied species had a different shape (Figures 1E–H, M–T) and size (Figure 2). In *L. japonicus* nodules, bacteroids were rod-shaped (Figures 1H,P,T), and were double the size of free-living bacteria (Figure 2). Bacteroids in nodules of *P. vulgaris* and *G. max* were bigger than the bacteroids of *L. japonicus* (Figure 2). In both *P. vulgaris* (Figures 1G,O,S) and *G. max* nodules (Figures 1E,M,Q), bacteroids exhibited a rod-shape. However, some bacteroids in *G. max* nodules were elongated (Supplementary Figure 1A) or elongated-branched (Supplementary Figures 1B,C). The most striking increase in bacteroid size was observed in *G. soja* nodules, which were about 5-fold longer than bacteria (some of them reached about 17 μm) (Figure 2) and had an elongated or elongated-branched shape (Figures 1F,N,R and Supplementary Figure 1F).

In the nodules of all studied species, both symbiosomes containing a single bacteroid and symbiosomes containing several bacteroids (multibacteroid symbiosomes) were found in infected cells (Figures 1M,O,P and Supplementary Figure 1E). The multibacteroid symbiosomes were formed as a result of the division of bacteroids (Supplementary Figure 1D).

The symbiosomes in infected cells in nodules of the studied species were randomly distributed. Symbiosomes in infected cells of *P. vulgaris* and *L. japonicus* nodules were more densely packed (Figures 1K,L) compared with symbiosomes of the infected cells of *G. max* (Figure 1I) and *G. soja* nodules (Figure 1J and Supplementary Video 1).

### Microtubule Organization in Meristematic Cells

**FIGURE 3:**
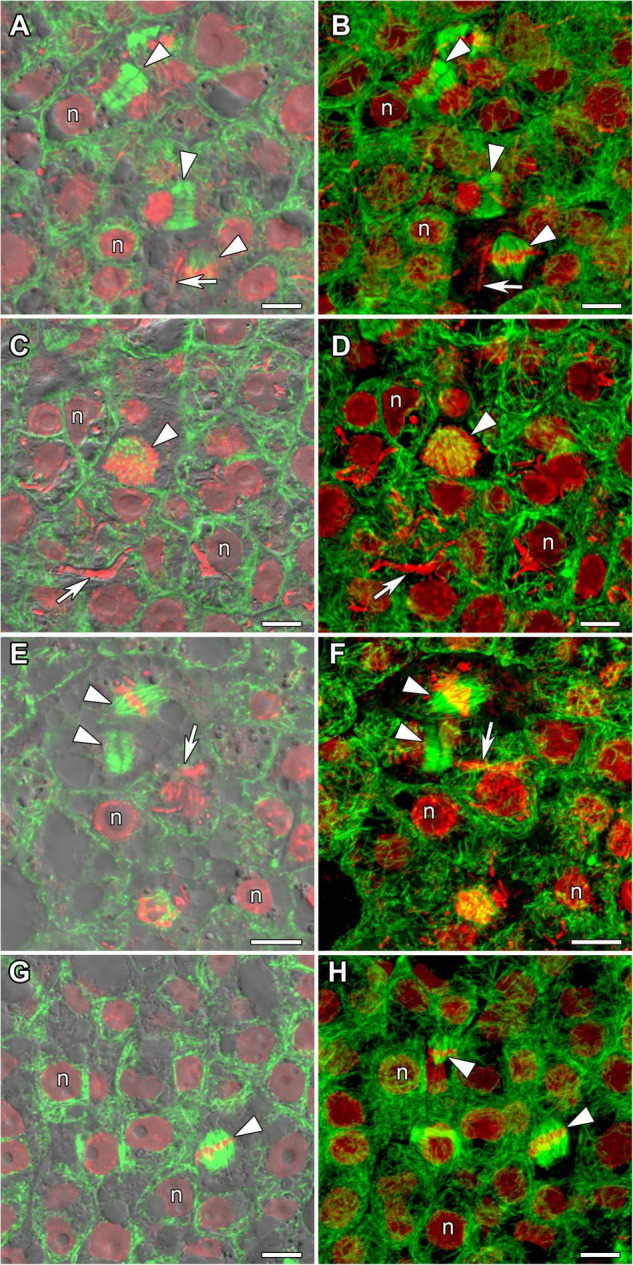
Microtubule organization in meristematic cells of *G. max* **(A,B)**, *G. soja* **(C,D)**, *P. vulgaris* **(E,F)**, and *L. japonicus* **(G,H)** nodules. n, nucleus; triangles indicate mitotic figures, arrows indicate infection threads. Confocal laser scanning microscopy of **(A–F)** 50 μm and **(G,H)** 35 μm longitudinal vibratome sections. Immunolocalization of tubulin (microtubules), green channel; DNA staining with propidium iodide (nuclei and bacteria), red channel. **(A,C,E,G)** Merged images of a single optical section of differential interference contrast and green and red channels. **(B,D,F,H)** Maximum intensity projections of 30 optical sections in green and red channels. Scale bar = 10 μm.

Determinate nodules are characterized by the absence of persistent meristems. In all analyzed species meristematic cells were visible in incipient 10-day-old nodules only (Figure 3). The infection threads reached these cells. Endoplasmic microtubules in meristematic cells were involved in the formation of mitotic spindles (Figure 3) and preprophase bands (Figures 3G,H). A dense network of randomly organized cortical microtubules formed irregular patterns (Figure 3). Perinuclear microtubules were randomly arranged around the nucleus and formed a dense network (Supplementary Video 2).

In developing 14-day-old nodules, some cells continued to divide among infected cells. In *G. max* nodules, cell division occurred in the center (Supplementary Figures 2A,B) as well as at the periphery of the nodule among maturating infected cells and parenchymal cells (Supplementary Figure 2C). In nodules of *G. soja*, mitoses were spread throughout the nodule, likely among uninfected cells (Supplementary Figures 3A,B). In nodules of *P. vulgaris*, mitoses were observed at the periphery of the nodule (Supplementary Figures 3C,D). In nodules of *L. japonicus*, dividing cells were observed only in incipient nodules.

### Microtubule Organization in Young Infected Cells

**FIGURE 4:**
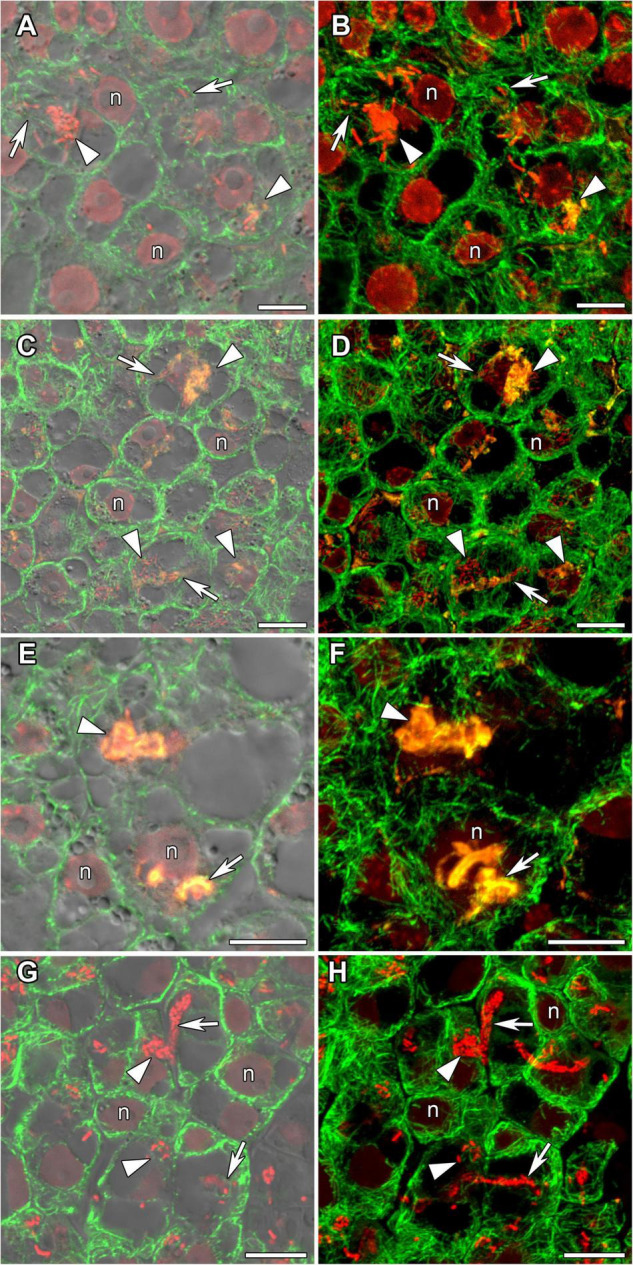
Cortical and endoplasmic microtubule organization in infected cells of infection zone in *G. max* **(A,B)**, *G. soja* **(C,D)**, *P. vulgaris* **(E,F)**, and *L. japonicus* **(G,H)** nodules. n, nucleus; triangles indicate infection droplets; arrows indicate infection threads. Confocal laser scanning microscopy of **(A–F)** 50 μm and **(G,H)** 35 μm longitudinal vibratome sections. Immunolocalization of tubulin (microtubules), green channel; DNA staining with propidium iodide (nuclei and bacteria), red channel and **(A–F)** immunolocalization of MAC265 (infection droplets), yellow channel. **(A,C,E)** Merged images of a single optical section of differential interference contrast and green, red, and yellow channels. **(G)** Merged images of a single optical section of differential interference contrast and green, and red channels. **(B,D,F)** Maximum intensity projections of **(B,D)** 40 and **(F)** 30 optical sections in green, red, and yellow channels. **(H)** Maximum intensity projections of 40 optical sections in green and red channels. Scale bar = 10 μm.

In incipient nodules after active cell division, cells began to be infected with released bacteria. In these infected cells the organization of cortical and endoplasmic microtubules was similar in nodules of all studied species (Figure 4). Cortical microtubules were oriented at different angles and formed an irregular pattern. Endoplasmic microtubules passed along infection threads and formed a dense network around infection droplets.

### Microtubule Organization in Uninfected Cells

**FIGURE 5:**
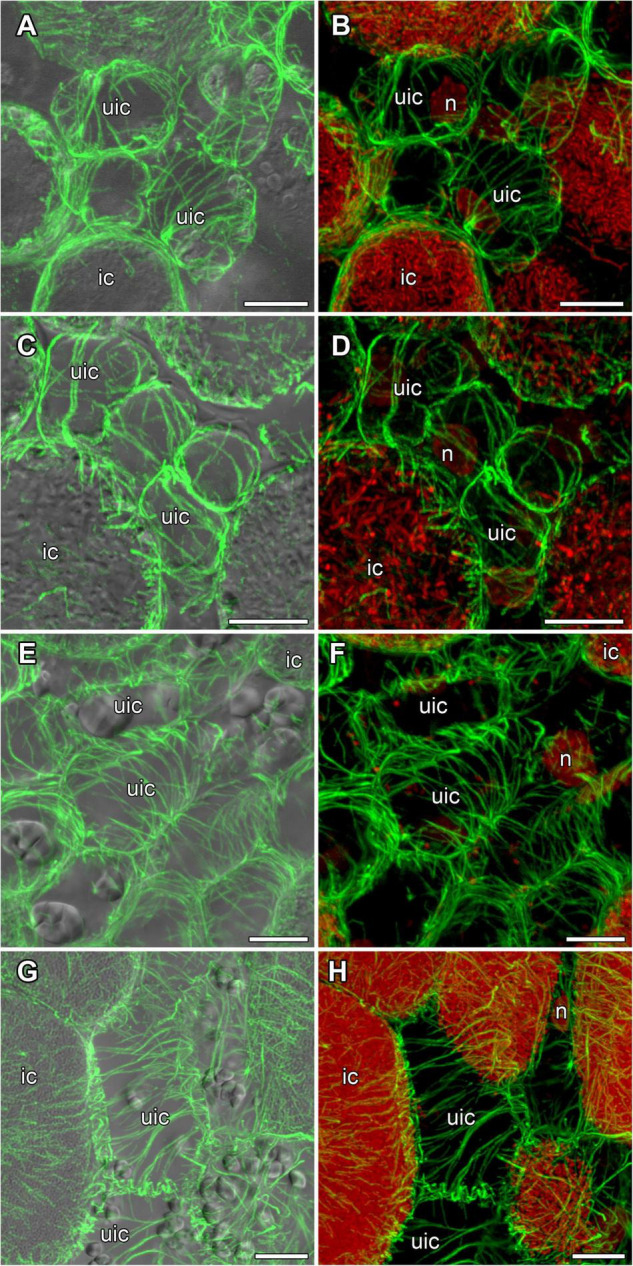
Cortical microtubule organization in uninfected cells of *G. max* **(A,B)**, *G. soja* **(C,D)**, *P. vulgaris* **(E,F)**, and *L. japonicus* **(G,H)** nodules. ic, infected cell; uic, uninfected cell; n, nucleus. Confocal laser scanning microscopy of **(A–F)** 50 μm and **(G,H)** 35 μm longitudinal vibratome sections. Immunolocalization of tubulin (microtubules), green channel; DNA staining with propidium iodide (nuclei and bacteria), red channel. **(A,C,E,G)** Merged images of a single optical section of differential interference contrast and maximum intensity projection of optical sections in the green channel. **(B,D,F,H)** Maximum intensity projections of **(B)** 45, **(D)** 55, and **(F,H)** 50 optical sections in green and red channels. Scale bar = 10 μm.

**FIGURE 6:**
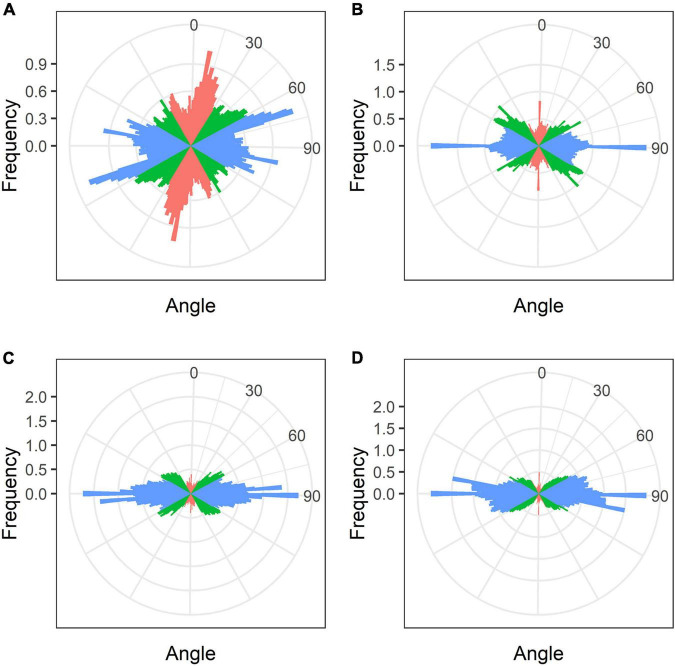
Quantitative analysis of cortical microtubule orientation in uninfected cells of *G. max* **(A)**, *G. soja* **(B)**, *P. vulgaris* **(C)**, and *L. japonicus* **(D)** nodules. Color indicates the class of angles of the microtubules relative to the longitudinal axis of the cell: red, axial (0–30°, 150–180°); green, oblique (30–60°, 120–150°); blue, transverse (60–120°).

In developing and mature (21-day-old) nodules of all four species, uninfected cells formed clusters (data not shown). In *G. max* nodules, uninfected cells were spherical (Figures 5A,B) and in *G. soja* nodules they were more or less spherical (Figures 5C,D), whereas in *L. japonicus* and *P. vulgaris* nodules, uninfected cells were elongated (Figures 5E–H). In uninfected cells only cortical microtubules were observed (Figure 5). In nodules of *G. max* and *G. soja*, cortical microtubules were organized at different angles and formed an irregular pattern (Figures 5A–D). Quantitative analysis revealed a roughly equal distribution of axial, oblique, and transverse microtubules in uninfected cells of *G. max* (Figure 6A and Supplementary Figure 4A). Uninfected cells of *G. soja* had fewer axial microtubules compared with *G. max* cells (Figure 6B and Supplementary Figure 4A). In uninfected cells of *L. japonicus* and *P. vulgaris* nodules, cortical microtubules formed a regular pattern (Figures 5E–H). The portions of axial and oblique microtubules in these nodules were lower than in *G. max* and *G. soja* nodules (Figures 6C,D and Supplementary Figure 4A); on the contrary, the portion of transverse microtubules was greater than 50% (Supplementary Figure 4A).

### Microtubule Organization in Nitrogen-Fixing Cells

**FIGURE 7:**
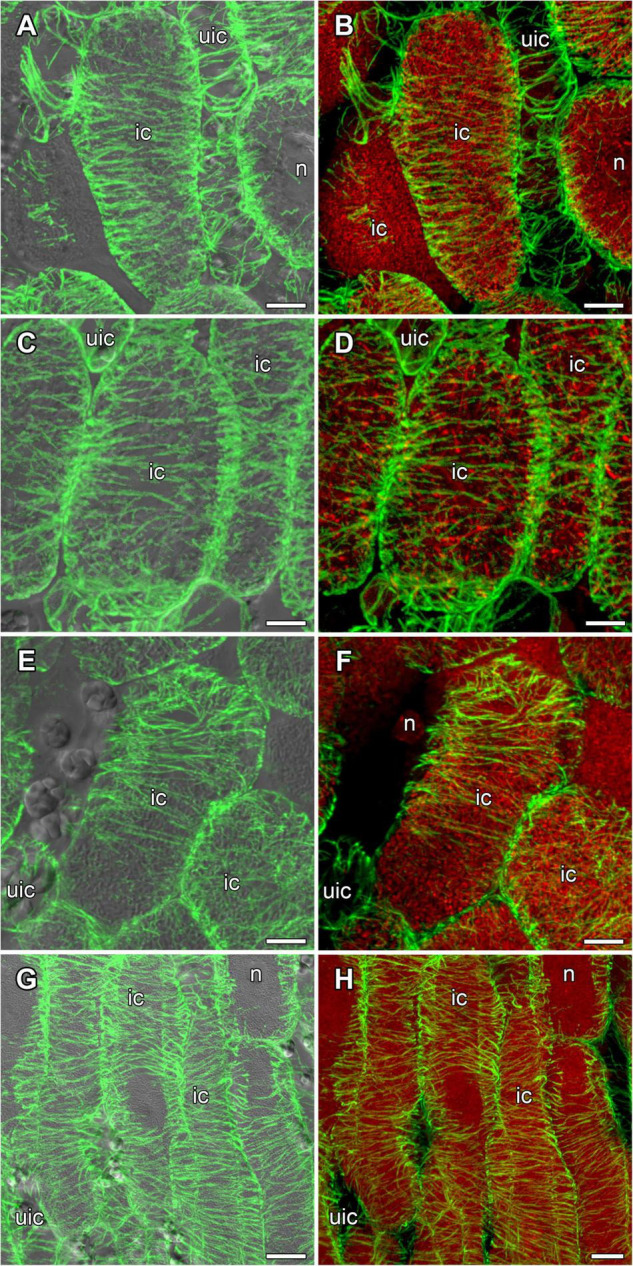
Cortical microtubule organization within infected cells of the nitrogen fixation zone in *G. max* **(A,B)**, *G. soja* **(C,D)**, *P. vulgaris* **(E,F)**, and *L. japonicus* **(G,H)** nodules. ic, infected cell; uic, uninfected cell; n, nucleus. Confocal laser scanning microscopy of **(A–F)** 50 μm and **(G,H)** 35 μm longitudinal vibratome sections. Immunolocalization of tubulin (microtubules), green channel; DNA staining with propidium iodide (nuclei and bacteria), red channel. **(A,C,E,G)** Merged images of a single optical section of differential interference contrast and maximum intensity projection of optical sections in the green channel. **(B,D,F,H)** Maximum intensity projections of **(B,D)** 50, **(F)** 20 and **(H)** 55 optical sections in green and red channels. Scale bar = 10 μm.

**FIGURE 8:**
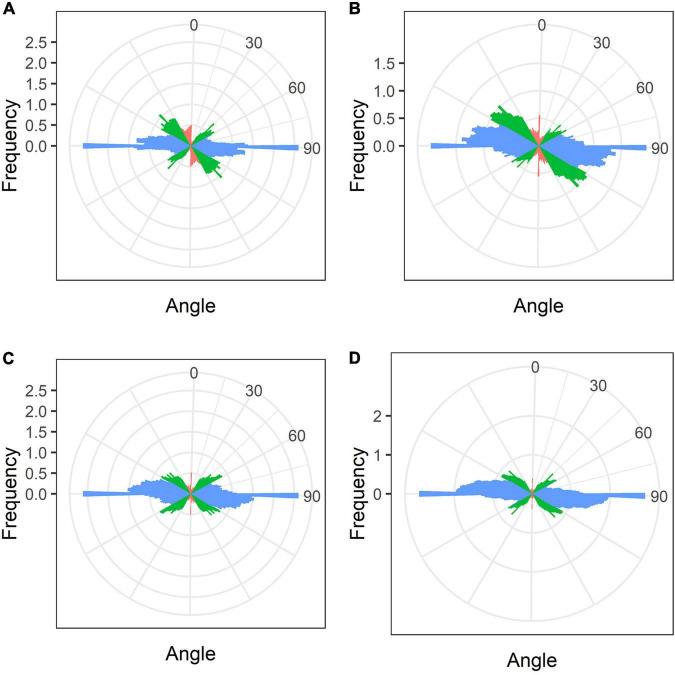
Quantitative analysis of cortical microtubule orientation in infected cells of the nitrogen fixation zone of *G. max* **(A)**, *G. soja* **(B)**, *P. vulgaris* **(C)**, and *L. japonicus* **(D)** nodules. Color indicates the class of angles of the microtubules relative to the longitudinal axis of the cell: red, axial (0–30°, 150–180°); green, oblique (30–60°, 120–150°); blue, transverse (60–120°).

In mature nodules of all studied species, nitrogen-fixing cells were elongated (Figure 7). Both cortical and endoplasmic microtubules were distinguished. Cortical microtubules were arranged parallel to each other and perpendicular to the longitudinal axis, forming a regular pattern in all studied species (Figure 7). However quantitative analysis showed that the studied species varied in the number of different types of microtubules. The smallest number of transverse microtubules (and the largest axial) was in *G. max* (Figure 8A and Supplementary Figure 4B), and the largest number of transverse microtubules (and the smallest axial) was in *L. japonicus* (Figure 8D and Supplementary Figure 4B). In *G. soja* and *P. vulgaris*, the number of transverse and axial microtubules occupied an intermediate value (Figures 8B,C and Supplementary Figure 4B).

**FIGURE 9:**
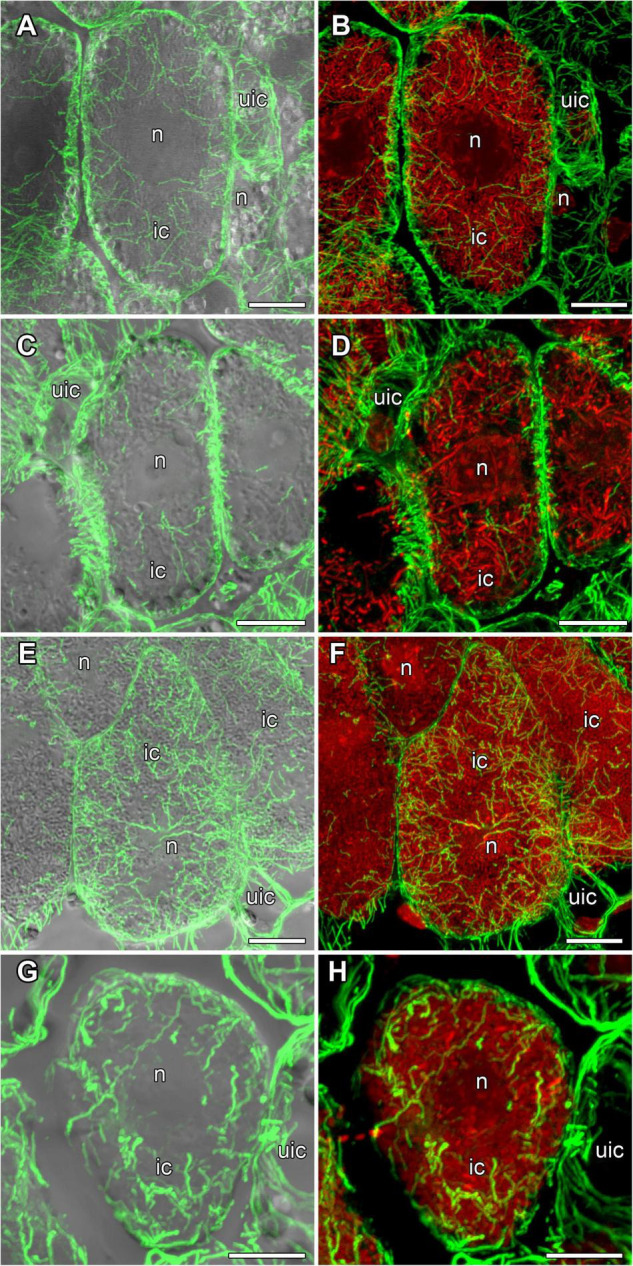
Endoplasmic microtubule organization in infected cells of the nitrogen fixation zone in *G. max* **(A,B)**, *G. soja* **(C,D)**, *P. vulgaris* **(E,F)**, and *L. japonicus* **(G,H)** nodules. ic, infected cell; uic, uninfected cell; n, nucleus. Confocal laser scanning microscopy of **(A–F)** 50 μm and **(G,H)** 35 μm longitudinal vibratome sections. Immunolocalization of tubulin (microtubules), green channel; DNA staining with propidium iodide (nuclei and bacteria), red channel. **(A,C,E,G)** Merged images of a single optical section of differential interference contrast and maximum intensity projection of optical sections in the green channel. **(B,D,F,H)** Maximum intensity projections of **(B,D)** 30, **(F)** 40, and **(H)** 30 optical sections in green and red channels. Scale bar = 10 μm.

In nodules of all four legume species, endoplasmic microtubules formed networks located among symbiosomes in infected cells (Figure 9). Thick long bundles were barely branched and passed from the center part of the cell to the periphery (Supplementary Video 3). The density of endoplasmic microtubules in nitrogen-fixing cells of nodules *G. max* (Figures 9A,B) and *P. vulgaris* (Figures 9E,F) and in cells of nodules *G. soja* (Figures 9C,D) and *L. japonicus* (Figures 9G,H) looked similar.

### Quantitative Analysis of Endoplasmic Microtubules in Nitrogen-Fixing Cells

**FIGURE 10:**
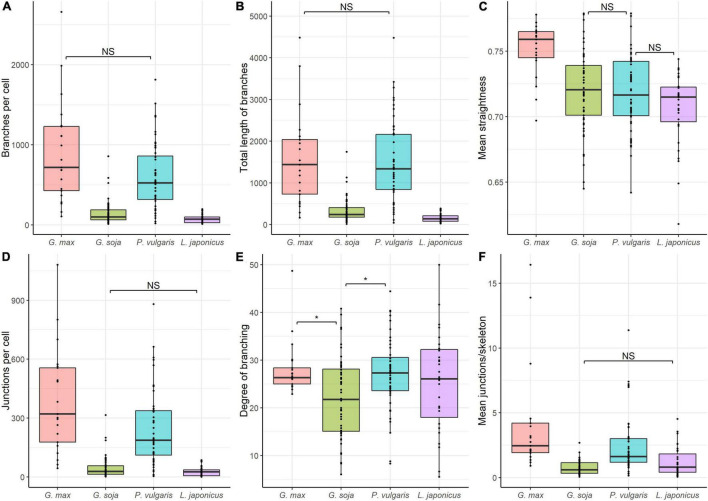
Quantitative analysis of tubulin microtubule organization in nitrogen-fixing cells of *G. max* (*n* = 21), *G. soja* (*n* = 56), *P. vulgaris* (*n* = 48), and *L. japonicus* (*n* = 31) nodules. **(A)** Total number of branches per cell, number of all detected microtubules. **(B)** Total length of branches per cell, the total length of all detected microtubules per cell. **(C)** Mean straightness index of detected microtubules per cell, the Euclidian distance between the starting and ending point of each branch divided by its full length. **(D)** Total number of junctions per cell. **(E)** Degree of branching per cell, number of skeletons (sets of branches, connected together) with more than one branch divided by the total number of skeletons in the image. **(F)** Mean number of junctions per skeleton, average number of branching points across all skeletons in the cell. Dots indicate analyzed images of individual cells. Pairwise comparisons were conducted using Tukey’s range test, NS indicates not significant differences (*p* > 0.05), otherwise differences are significant (*p* < 0.05).

Quantitative analysis revealed similarities in the mean number of branches per cell (Figure 10A), and total length of branches (Figure 10B) between endoplasmic microtubules in nitrogen-fixing cells of *G. max* and *P. vulgaris* nodules. The nitrogen-fixing cells of *G. soja* and *L. japonicus* nodules had a similar number of junctions per cell (Figure 10D) and mean number of junctions per skeleton (Figure 10F). The degree of branching was similar for all four species. However, there were statistical differences between *G. max* and *G. soja* and between *P. vulgaris* and *G. soja* (Figure 10E). These results demonstrated that the density of endoplasmic microtubules in nitrogen-fixing cells of nodules of *G. max* and *P. vulgaris* and in cells of nodules of *G. soja* and *L. japonicus* were similar. Endoplasmic microtubules in nitrogen-fixing cells of *G. max* were characterized by the highest mean straightness compared with the other three species (Figure 10C). At the same time cells of *G. soja* and *L. japonicus* were characterized by a similar mean number of junctions per skeleton (Figure 10F).

## Discussion

### Bacteroid Morphology

The shape and size of bacteroids are controlled by the host plant (Terpolilli et al., 2012). Generally, elongated and branched bacteroids are characteristic of indeterminate nodules, and rod-shaped ones of determinate nodules (Oono et al., 2010; Montiel et al., 2017). However, in *C. arietinum* and *Glycyrrhiza uralensis* indeterminate nodules, bacteroids are spherical and swollen, respectively (Montiel et al., 2016; Kitaeva et al., 2021; Tsyganova et al., 2021). In the current study in *L. japonicus* nodules, bacteroids were similar to free-living bacteria by shape, but their size showed a twofold increase (Figures 1D,H,P,T, 2). It is important to note, that originally only a 20% increase in size in comparison with free-living bacteria was described for bacteroids in *L. japonicus* nodules (Szczyglowski et al., 1998). However, more latterly, a significant increase in bacteroid size in *L. japonicus* nodules has been shown (Suganuma et al., 2003). The rod-shaped form of bacteroids in infected cells of *L. japonucus* nodules has been previously described (Pankhurst et al., 1979; Imaizumi-Anraku et al., 1997; Szczyglowski et al., 1998; Suganuma et al., 2003; Regus et al., 2017). However, upon inoculation of *L. angustissimus* L., *L. pedunculatus* Car., and *L. tenuis* Waldst. Et Kit. with different rhizobia species, bacteria differentiated into spherical, swollen, or rod-shaped bacteroids (Craig and Williamson, 1972; Craig et al., 1973). Moreover, when *L. japonicus* was inoculated with the *R. etli* strain CE3, a significant part of symbiosomes contained a single bacteroid with an elongated and even branching shape (Banba et al., 2001).

On the contrary, upon inoculation of *P. vulgaris* and *G. max* plants with various species and strains of rhizobia, differentiated bacteroids were uniformly rod-shaped (Werner and Mörschel, 1978; Studer et al., 1992; Cermola et al., 1994; Moris et al., 2005; Arthikala et al., 2014), which has been confirmed by studies using scanning electron microscopy (Tu, 1975, 1977). According to our data, the bacteroids in nodules of these species were morphologically similar upon inoculation of *P. vulgaris* with the *R. leguminosarum* bv. *phaseoli* RCAM2624 and *G. max* with *B. liaoningense* RCAM04656 (Figures 1E,G,M,O,Q,S). Measurements of the length of the bacteroids in nodules of these species showed them to be similar and equal to approximately 3.3 μm and 3.5 μm (Figure 2) for nodules of *P. vulgaris* and *G. max*, respectively. In previous studies, the length of bacteroids in *P. vulgaris* nodules upon inoculation with *R. etli* was 1.79 μm (Moris et al., 2005), while the length of bacteroids in *G. max* nodules upon inoculation with *B. japonicum* was different in various studies: 3–5 μm (Bergersen and Briggs, 1958) and about 2.5 μm (Montiel et al., 2016). The observed variation of bacteroid length can be explained by the difference in the rhizobia species infecting the nodules.

It was especially striking that bacteroids in the symbiosis between *G. soja* and *B. liaoningense* RCAM04656 significantly increased in size, reaching 5.5 μm (some of them reached about 17 μm) (Figures 1F,N,R, 2). Unfortunately, studies of *G. soja* bacteroid morphology are limited. For example, in *G. soja* nodules infected with *S. fredii*, bacteroids were spherical, swollen, and rod-shaped (Temprano-Vera et al., 2018), while upon inoculation with *B. diazoefficiens* sp. nov. USDA110 they were rod-shaped or elongated (Muñoz et al., 2016). These differences can be explained by the fact that different species of rhizobia were used for plant inoculation.

### Symbiosomes

In this study, in the nodules of all studied species, both symbiosomes with a single bacteroid and multibacteroid symbiosomes were presented in infected cells (Figures 1M–P and Supplementary Figure 1E). Previously it was shown that in mature determinate nodules, symbiosomes usually contain two or more bacteroids (Lodwig et al., 2005; Oono et al., 2010) and their number depends on the conditional stage of development (early, intermediate, or late), and all three stages can be present simultaneously in nodules. For example, in young infected cells of *P. vulgaris* (Bal et al., 1982; Cermola et al., 2000) and *G. max* (Bergersen and Briggs, 1958; Werner and Mörschel, 1978; Reagan et al., 2017) nodules, symbiosomes contain a single bacteroid, which was confirmed by focused ion beam-scanning electron microscopy and 3D reconstruction (Reagan et al., 2017). With age, after several rounds of division of bacteroids, their number can be increased up to 20 (Muñoz et al., 2016). The increase in bacteroid number in mature symbiosomes can be also caused by the fusion of symbiosomes containing a single bacteroid (Fedorova et al., 1999; Cermola et al., 2000). In 2–3-week-old nodules (nodules of this age were analyzed in this study) both single and multibacteroid symbiosomes can be observed (Studer et al., 1992; Imaizumi-Anraku et al., 1997; Cermola et al., 2000; Ott et al., 2009; Arthikala et al., 2014; Temprano-Vera et al., 2018).

### Microtubular Organization

In the current study, we performed a comparative analysis of the organization of the tubulin cytoskeleton in determinate nodules of four legume species (Table 1), which should complement our previous studies of the organization of microtubules in indeterminate nodules of six legume species (Kitaeva et al., 2016, 2021; Tsyganova et al., 2021). The study included nodules of various ages, which made it possible to observe the dynamics of changes in the organization of the tubulin cytoskeleton. At the same time, both the patterns of cortical microtubules and endoplasmic ones were analyzed. When analyzing endoplasmic microtubules, special attention was paid to their interaction with infection structures in the cell.

**TABLE 1:** Comparative analysis of microtubular patterns in determinate nodules of *G. max*, *G. soja*, *P. vulgaris*, *L. japonicus*, and indeterminate nodules.

Cell type	Type of microtubules	*Glycine max*	*Glycine soja*	*Phaseolus vulgaris*	Lotus japonicus*	Indeterminate nodules**
Meristematic	*Cortical*	Irregular				Irregular
	
	*Endoplasmic*	Mitotic spindles, preprophase bands, perinuclear				Mitotic spindles, preprophase bands, perinuclear

Young infected	*Cortical*	Irregular				Irregular
	
	*Endoplasmic*	A network around infection threads and droplets				A network around infection threads and droplets

Uninfected	*Cortical*	Irregular***		Regular		**Regular**
	
	*Endoplasmic*	Unidentified				Unidentified

Nitrogen-fixing	*Cortical*	**Regular**				**Irregular**
	
	*Endoplasmic*	A network among symbiosomes; **irregular**				A network among symbiosomes; irregular, **regular**, and intermediate

*The current study.

**Based on Kitaeva et al., 2016, 2021; Tsyganova et al., 2021.

***Differences observed between microtubular patterns in determinate and indeterminate nodules are shown in bold.

Despite the transient character of meristem functioning in determinate nodules, in incipient (10-day-old) nodules of all analyzed legume species, numerous meristematic cells were observed (Figure 3). Endoplasmic microtubules were involved in the formation of mitotic spindles and preprophase bands whereas cortical microtubules formed irregular patterns (Table 1 and Supplementary Video 2). The organization of both cortical and endoplasmic microtubules was similar to that described for meristematic cells of indeterminate nodules (Kitaeva et al., 2016, 2021; Tsyganova et al., 2021) and root meristem cells (Baluška et al., 1992; Adamakis et al., 2010). In developing nodules, in *G. max*, *G. soja*, and *P. vulgaris*, some mitoses were still visible (Supplementary Figures 2, 3). In *G. soja* mitotic activity was observed among uninfected cells that likely led to the formation of clusters of uninfected cells (see below).

In young, infected cells of all studied legume species, cortical microtubules formed irregular patterns and endoplasmic ones were associated with infection threads and infection droplets (Figure 4 and Table 1). These patterns were similar to those observed in indeterminate nodules (Kitaeva et al., 2016, 2021; Tsyganova et al., 2021). This indicates that the development of infection in nodules shares common mechanisms in both determinate and indeterminate nodules.

Analysis of the orientation of cortical microtubules in uninfected cells revealed striking differences between *G. max* and *G. soja* nodules on the one hand, and *P. vulgaris* and *L. japonicus* nodules, on the other. Uninfected cells in *G. max* (Figures 5A,B and Table 1) and *G. soja* (Figures 5C,D and Table 1) nodules were characterized by an irregular pattern, while uninfected cells in *P. vulgaris* (Figures 5E,F and Table 1) and *L. japonicus* (Figures 5G,H and Table 1) nodules were characterized by a regular pattern. The observed differences were confirmed by quantitative analysis that revealed predominant transverse orientation of microtubules in uninfected cells of *P. vulgaris* and *L. japonicus* (Figures 6C,D). The observed differences in the patterns of cortical microtubules suggest that they lead to various types of growth (isodiametric and anisotropic), which is reflected in the form of uninfected cells. In *G. max* and *G. soja*, they are spherical, and in *P. vulgaris* and *L. japonicus*, they are elongated. Early investigation revealed similar irregular patterns of cortical microtubules in uninfected cells in nodules of different age in *G. max* (Whitehead et al., 1998). In indeterminate nodules, it was previously demonstrated that the cortical microtubules of uninfected cells changed from an irregular pattern in the infection zone to a regular one in the nitrogen fixation zone (Kitaeva et al., 2016, 2021; Tsyganova et al., 2021). Thus, the pattern of uninfected cells formed by cortical microtubules in nodules of *G. max* and *G. soja* seems to be unique for the *Glycine* genus and is not linked to nodule type.

It should be noted that, in all four species, uninfected cells were grouped into clusters. The formation of uninfected cells in groups and rows was previously described for *G. max* nodules (Selker and Newcomb, 1985). It is likely that such an arrangement will ensure contact of small, uninfected cells with large, infected cells and facilitate the transfer of ammonia into them. On the other hand, it is possible that the formation of clusters of uninfected cells is associated with the production and transport of ureids, which are the product of nitrogen assimilation in determinate nodules (Pate et al., 1980; Newcomb and Tandon, 1981; Newcomb et al., 1985).

In mature nodules of all four legume species, the cortical microtubules in infected cells formed well pronounced regular patterns (Figure 7 and Table 1) that were confirmed by quantitative analysis (Figure 8). Nevertheless, some variation in the number of microtubules of different orientations was observed between species (Supplementary Figure 4B). A regular pattern has been previously observed in developing (15-day-old) *G. max* nodules; in mature nodules (42–49-day-old), which were significantly older than the mature nodules analyzed in this study (21-day-old); the regular pattern was retained only in certain regions of the cell (Whitehead et al., 1998). The observed regular pattern of cortical microtubules in infected cells in determinate nodules is strikingly different from the irregular pattern characteristic of indeterminate nodules (Kitaeva et al., 2016, 2021; Tsyganova et al., 2021). The revealed differences mean that infected cells in determinate nodules use anisotropic growth to increase the cell volume and accommodate symbiosomes, while isodiametric growth is used for these purposes in indeterminate nodules. The reason for the identified differences is fascinating, but is currently unclear. It is unlikely to be associated with the morphology of bacteroids, since although in *G. max*, *P. vulgaris*, and *L. japonicus* bacteroids are less differentiated than bacteroids in indeterminate nodules, in *G. soja* there was a significant increase in the size of bacteroids compared to bacteria, while infected cells of *G. soja* were also characterized by anisotropic growth. Taking into consideration the fact that determinate nodules within the Papilionoideae subfamily appeared at an earlier stage of evolution than indeterminate nodules (Ren, 2018), it can be assumed that the transition of infected cells to isodiametric growth was an adaptation providing an evolutionary advantage of indeterminate nodules. It is possible that, topologically, the distribution of symbiosomes in an infected cell in the form of a sphere, which is created by isodiametric growth, is facilitated in comparison with cylindrical infected cells resulting from anisotropic growth. It is also important to note that, in infected cells, the central vacuole is absent in determinate nodules, while in indeterminate nodules a large vacuole occupies a central position in the cell (Newcomb, 1976). Nevertheless, in order to confirm the universality of the identified patterns of cortical microtubules for infected cells in determinate and indeterminate nodules, it is necessary to analyze the organization of the tubulin cytoskeleton in a greater number of legume species. To date, of the six legume species forming indeterminate nodules, for which the analysis of the organization of the tubulin cytoskeleton was carried out, five belong to the same Vicioid clade (Wojciechowski et al., 2004). Future analyses should include species representing different clades of legumes.

In the infected cells in the nodules of all four studied species, a well-developed network of endoplasmic microtubules, forming an irregular pattern and located between chaotically distributed symbiosomes, was observed (Figure 9 and Table 1). However, surprisingly, the visual network of endoplasmic microtubules was observed to be denser in *G. max* and *P. vulgaris* infected cells than in *G. soja* and *L. japonicus* cells. The observed differences were confirmed by quantitative analysis (Figure 10). Previously, we observed an irregular and regular pattern of endoplasmic microtubules in infected cells of various legume species, which coincided with an ordered or disordered arrangement of symbiosomes, respectively (Kitaeva et al., 2016, 2021; Tsyganova et al., 2021). Nevertheless, in mature determinate nodules, symbiosomes containing several bacteroids are present and, accordingly, their size is significantly larger than the size of the bacteroids themselves, as assessed in this study (Figure 2). It would be interesting to evaluate the size and shape of symbiosomes in determinate nodules and relate them to the pattern of endoplasmic microtubules.

## Conclusion

Thus, the similarity of the organization of endoplasmic microtubules involved in the development of infection threads and infection droplets in the cells of indeterminate and determinate nodules revealed the commonality of the role of tubulin cytoskeleton in the development of infection structures in nodules of both types. Notable differences were revealed in the organization of cortical microtubules in infected cells between indeterminate and mature determinate nodules, which were manifested in the isodiametric growth of infected cells in an indeterminate nodule and anisotropic growth in a determinate nodule. The fact that within the Papilionoideae subfamily determinate nodules appeared in evolution earlier than indeterminate ones raises an intriguing question regarding the possible advantage of isodiametric growth of infected cells over anisotropic for their accommodation of numerous symbiosomes. Further research should address this question. However, it is necessary to analyze the organization of the tubulin cytoskeleton in a larger number of legume species in order to confirm the revealed patterns of cortical microtubule organization in infected cells of determinate and indeterminate nodules.

## Data Availability Statement

The raw data supporting the conclusions of this article will be made available by the authors, without undue reservation.

## Author Contributions

VT: conceptualization and writing—review and editing. AG and AT: electron microscopy studies. AK: immunolocalization of tubulin cytoskeleton and laser scanning confocal microscopy. PK and AS: quantitative analysis of tubulin cytoskeleton. AK and AT: writing—original draft preparation. All authors have read and agreed to the published version of the manuscript.

## Conflict of Interest

The authors declare that the research was conducted in the absence of any commercial or financial relationships that could be construed as a potential conflict of interest.

## Publisher’s Note

All claims expressed in this article are solely those of the authors and do not necessarily represent those of their affiliated organizations, or those of the publisher, the editors and the reviewers. Any product that may be evaluated in this article, or claim that may be made by its manufacturer, is not guaranteed or endorsed by the publisher.

## Supplementary Material

Supplementary Video 1 Organization of bacteroids in infected cells of mature *G. soja* nodules.

Supplementary Video 2 Organization of microtubules in meristematic cells of *G. max* nodules.

Supplementary Video 3 Organization of microtubules in infected cells of mature *P. vulgaris* nodules.
