# Supplementary material for: Tubulin Cytoskeleton Organization in Cells of Determinate Nodules
Source: Front Plant Sci. 2022 Apr 26;13:823183. doi: 10.3389/fpls.2022.823183 (PMC9087740; doi:10.3389/fpls.2022.823183)
Supplement: Supplementary file 1 [file Data_Sheet_1.pdf]

Supplementary Material

Tubulin Cytoskeleton Organization in Cells of Determinate Nodules

Anna B. Kitaeva[1], Artemii P. Gorshkov[1], Pyotr G. Kusakin[1], Alexandra R. Sadovskaya[2], Anna V. Tsyganova,[1] and Viktor E. Tsyganov[1,3]*

[1]Laboratory of Molecular and Cellular Biology, All-Russia Research Institute for Agricultural Microbiology, Saint Petersburg, Russia

[2]Saint Petersburg State University, Saint Petersburg, Russia

[3]Saint Petersburg Scientific Center RAS, Saint Petersburg, Russia

*** Correspondence:**
Viktor Tsyganov
vetsyganov@arriam.ru

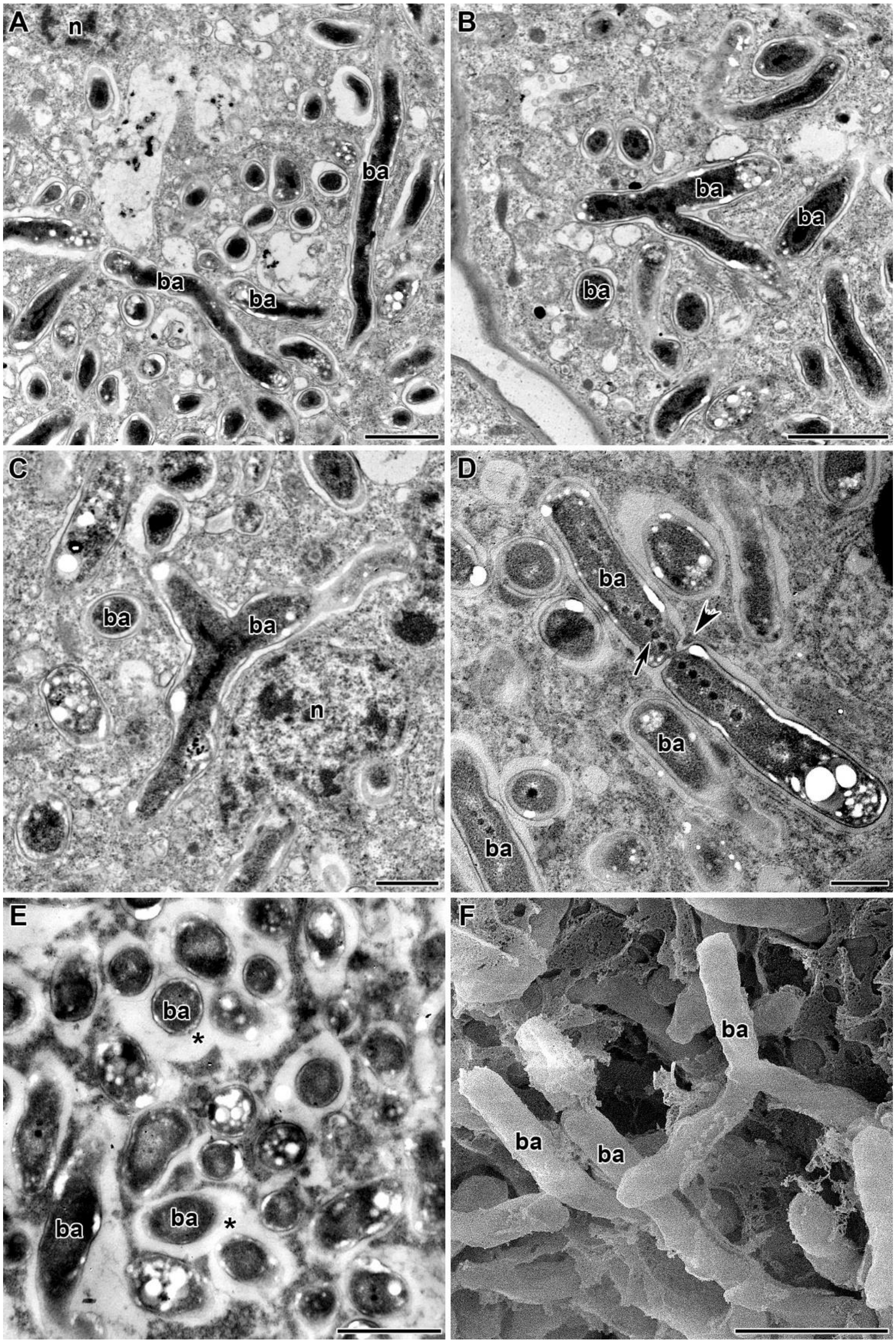

Supplementary Figure 1. Morphology of bacteroids and symbiosomes in *G. max* **(A–D)** and *G. soja* **(E, F)** nodules. ba, bacteroid; *, multibacteroid symbiosome; arrow indicates nucleoid; arrowhead indicates division. **(A)** Elongated bacteroid. (**B, C**) Elongated-branched bacteroids. **(D)** Division of bacteroid. **(E)** Multibacteroid symbiosomes. **(F)** Branched bacteroid. Scale bar **(A, B, D)** = 2 μm, **(C, E)** = 1 μm, **(D)** = 500 nm.

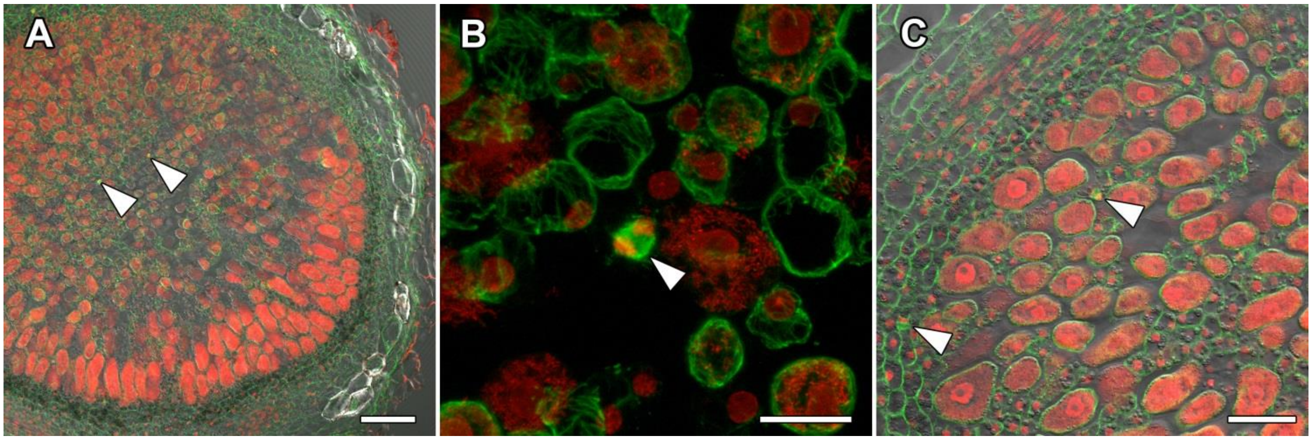

Supplementary Figure 2. Cell divisions in developing nodules of *G. max*. Mitoses in the center of the nodule **(A, B)**. Mitoses at the periphery of the nodule **(C)**. n, nucleus; ic, infected cell; uic, uninfected cell; arrowheads indicate mitotic figures. Confocal laser scanning microscopy of 50 µm longitudinal vibratome sections. Immunolocalization of tubulin (microtubules), green channel; DNA staining with propidium iodide (nuclei and bacteria), red channel. **(A, C)** Merged images of a single optical section of differential interference contrast, green and red channels. **(B)** Maximum intensity projections of 45 optical sections in green and red channels. Scale bar **(A)** =100 µm, **(B)** 20 = µm, **(C)** 50 = µm.

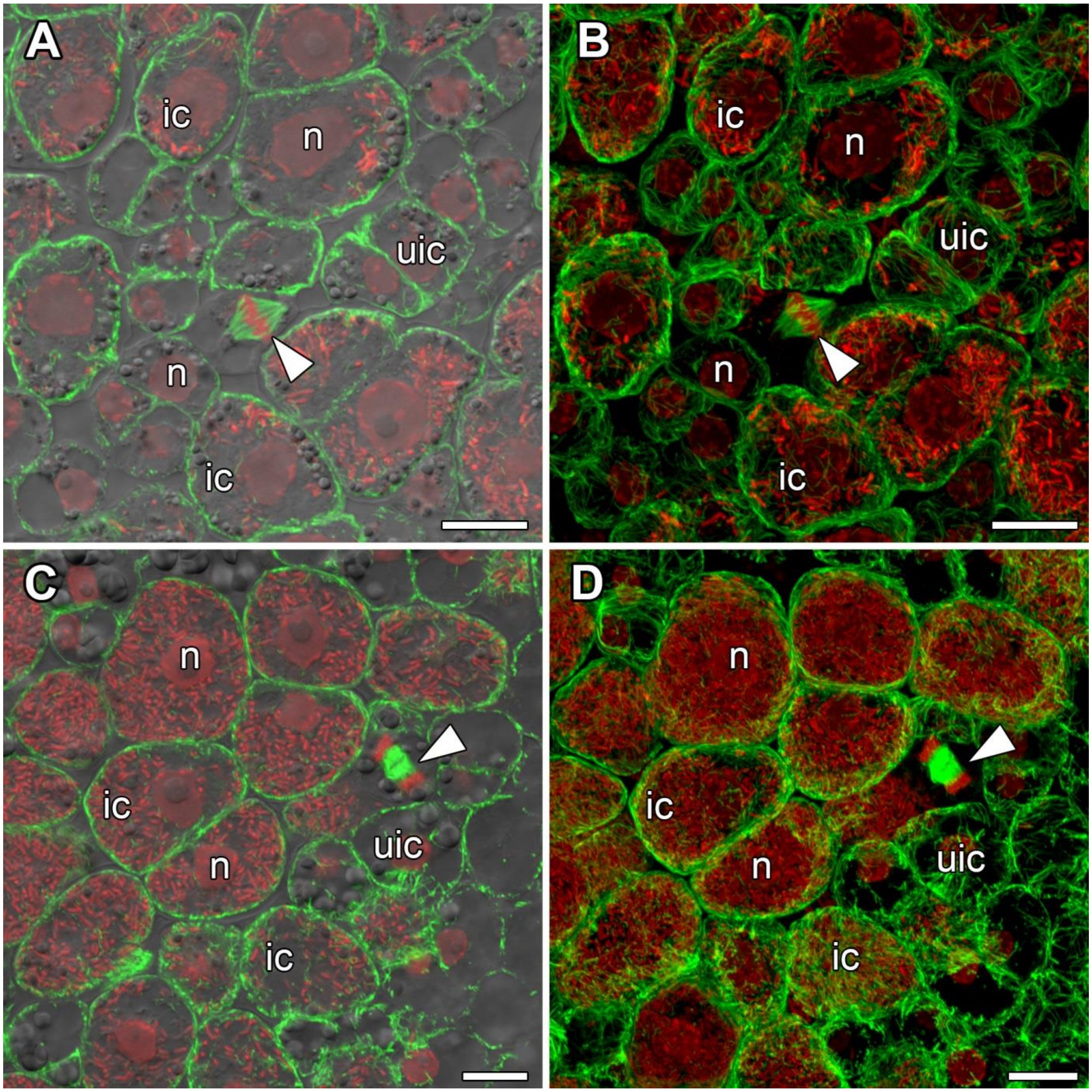

Supplementary Figure 3. Cell division in developing nodules of *G. soja* **(A, B)**, *P. vulgaris* **(C, D)**. ic, infected cell; uic, uninfected cell; n, nucleus. Confocal laser scanning microscopy of 50 µm longitudinal vibratome sections. Immunolocalization of tubulin (microtubules), green channel; DNA staining with propidium iodide (nuclei and bacteria), red channel. **(A, C)** Merged images of a single optical section of differential interference contrast and maximum intensity projection of optical sections in green and red channels. **(B, D)** Maximum intensity projections of 30 optical sections in green and red channels. Scale bar = 10 µm.

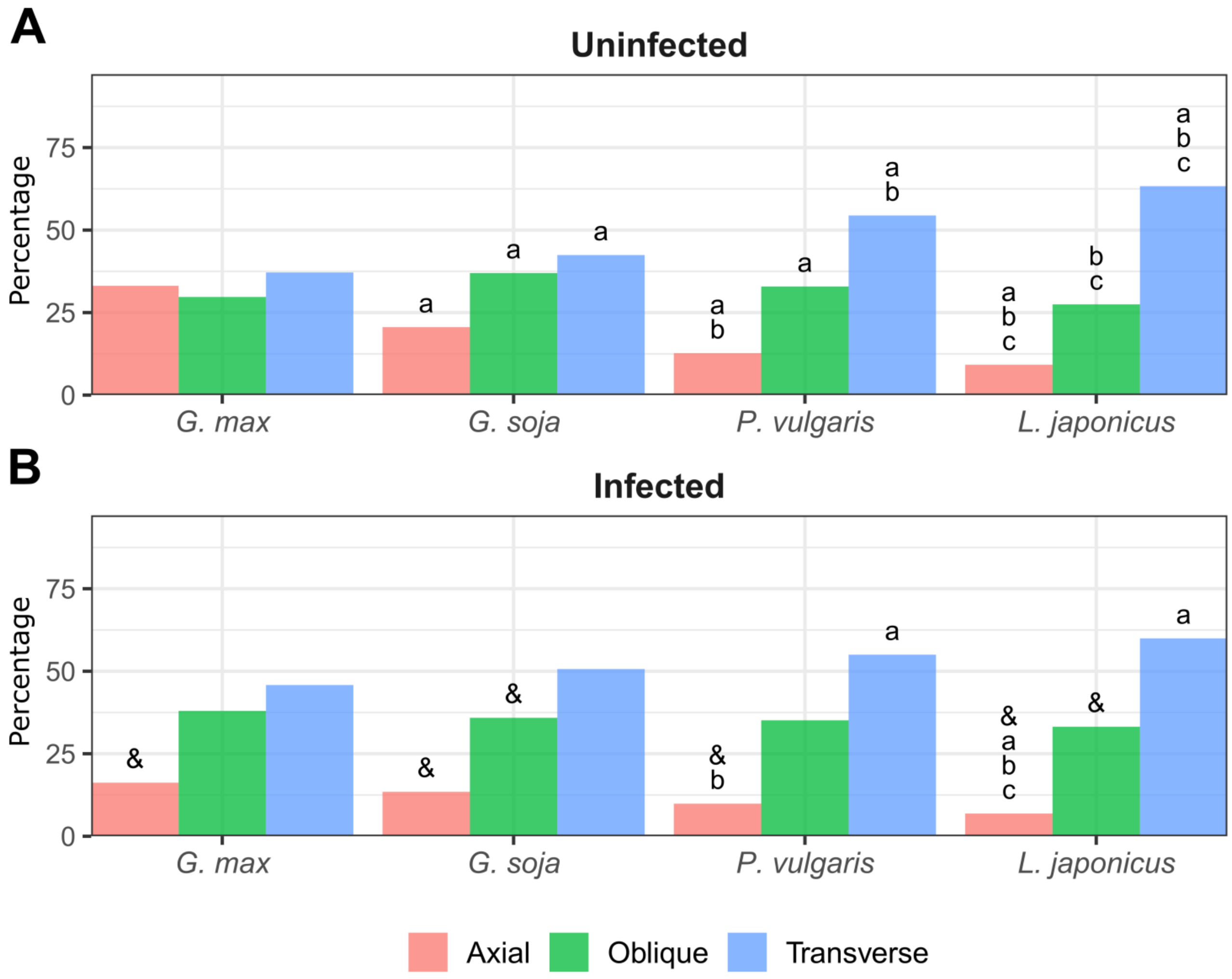

Supplementary Figure 4. The percentage of cortical microtubules of each class in uninfected **(A)** and infected **(B)** cells of *G. max*, *G. soja*, *P. vulgaris*, and *L. japonicus* nodules. Color indicates the different classes of microtubule orientation relative to the longitudinal axis of the cell. Letters indicate significant differences (Kruskal-Wallis and Dunn's tests, p > 0.05) of angle frequencies between studied species in the same class as follows: a, from *G. max*; b, from *G. soja*; c, from *P. vulgaris*, and indicates significant differences (Wilcoxon rank-sum test with BH correction for multiple comparisons, *p* > 0.05) of angle frequencies between uninfected and infected cells of the same species.
